# Data supporting the identification of compound for inhibition of survivin of colorectal cancer by using ingenuity pathway analysis of gene expression profiling of colorectal cancer tissues

**DOI:** 10.1016/j.dib.2015.05.017

**Published:** 2015-06-12

**Authors:** Yi-Chao Lee, Jun-Wei Lee, Chi-Chen Huang, Ming-Heng Wu, Kuen-Haur Lee

**Affiliations:** a The PhD Program for Neural Regenerative Medicine, College of Medical Science and Technology, Taipei Medical University, Taipei, Taiwan; b Institute of Biomedical Sciences, Academia Sinica, Taipei, Taiwan; c The PhD Program for Translational Medicine, College of Medical Science and Technology, Taipei Medical University, Taipei, Taiwan; d Graduate Institute of Cancer Biology and Drug Discovery, College of Medical Science and Technology, Taipei Medical University, 250 Wu-Hsing Street, Taipei 11031, Taiwan

## Abstract

The data in this article is related to the research article entitled, “Targeting of Multiple Oncogenic Signaling Pathways by Hsp90 Inhibitor Alone or in Combination with Berberine for Treatment of Colorectal Cancer” [1]. Overexpression of survivin induces resistance to various anticancer therapies such as chemotherapy and radiation therapy in colorectal cancer (CRC) cells. To determine significant correlations of biological functions/pathways with survivin, 4567 significant genes were analyzed from the GEO DataSet (GSE21815) of CRC and these were overlaid onto a global molecular network developed from information contained in the Ingenuity Pathway Analysis (IPA) database. The data here present the most significant disease and disordered biological functions, significant molecular/cellular functions and significant categories in physiological development/system functions which were associated with CRC. The top 10 canonical signaling pathways associated with CRC were categorize in order based on the level of statistical significance.

## Specifications table

**Table:** 

Subject area	Medicine, biology
More specific subject area	Molecular biology, Cancer biology
Type of data	Table
How data was acquired	Microarray data were obtained from the Gene Expression Omnibus (GEO) repository at the NCBI were analyzed using the Ingenuity pathway analysis (IPA) database
Data format	Analyzed
Experimental factors	None
Experimental features	Gene expression data of 132 samples of laser microdissected CRC tumors and nine normal colon controls were retrieved from the GEO DataSet under the accession number GSE21815 and imported into the IPA Tool to determine significant correlations of biological functions/pathways with survivin
Data source location	Taipei, Taiwan
Data accessibility	The data are supplied with this article

## Value of the data

• This data provides a comprehensive analysis of CRC patients gene expression profiling and identifies cell death and survival were the top significant molecular and cellular functional categories, EIF2 signaling, the protein ubiquitination pathway, and the eIF4/p70S6K signaling pathway were the most significant pathways in the upregulated CRC gene set.
• The data are useful for understanding the signaling pathway in the upregulated CRC gene set to be associated with survivin expression.
• This data may provide insight for determination the drug for combinational treatment of CRC.

## 1 Data, experimental design, materials and methods

### 1.1 Acquisition and processing of public microarray data

The public microarray data will be used here were obtained from the NNCBI GEO website (*GSE21815)*. Simple Omminus Format in Text (SOFT) files corresponding to the complete contents of GEO platform GPL6480 will be downloaded via FTP. The sequence information used to design this product was derived from a broad survey of well known sources such as RefSeq, Ensembl and Unigene. The resulting view of the human genome covers 41 K unique genes and transcripts which have been verified and optimized by alignment to the human genome assembly and by Agilent׳s Empirical Validation process. Microarray expression data for the GEO data set will be retrieved for 132 stages I to IV CRC and 9 normal control from the NCBI GEO (GSE21815).

### 1.2 Ingenuity pathway analysis (IPA)

Gene expression data of 132 samples of laser microdissected CRC tumors and 9 normal colon controls of patients with no colorectal neoplasm were retrieved from the GEO DataSet under the accession number GSE21815 and imported into the IPA Tool (Ingenuity H Systems, Redwood City, CA, USA; http://www.ingenuity.com). A fold difference of 2.0-fold (up-regulated) or 0.5-fold (down-regulated) was considered significant and was applied prior to pathway analyses. Based on the Ingenuity Knowledge Base different networks, biological processes and/or diseases were then algorithmically generated based on connectivity of genes within the datasets. Focus molecules were identified by IPA on the basis of highest connectivity. Comparisons will be performed between CRC group or control. The genes showing significant differences in expression levels between groups will be submitted to IPA for human diseases and disorders, molecular and cellular functions categories and pathway analysis.

The most significant disease and disordered biological functions associated with CRC-correlated genes were related to cancer (Table 1, upper panel). Cell death and survival were the top significant molecular and cellular functional categories (Table 1, middle panel). Connective tissue development and function were the most significant categories in physiological development and system function (Table 1, bottom panel). To gain further insights into the pathogenesis of CRC, we analyzed CRC-correlated genes to elucidate dominant canonical pathways. The top 10 canonical signaling pathways were categorized in order based on the level of statistical significance (Table 2). Results of the pathway analysis showed that EIF2 signaling, the protein ubiquitination pathway, and the eIF4/p70S6K signaling pathway were the most significant pathways in the upregulated CRC gene set. Hsp90 inhibitors, such as geldanamycin, 17-AAG and NVP-AUY922 have been demonstrated to induce the overexpression of survivin to enhance cell survival and chemotherapy resistance in HT-29 cells [2]. More, mTOR/p70S6K signaling has been identified to be the upstream regulator of survivin [3]. Thus, targeting the mTOR/p70S6K/survivin axis is thus an attractive strategy in developing therapeutic agents against cancer cells with chemoresistance.

## Figures and Tables

**Table 1:** Biological functions associated with CRC.

**Network**	**Top functions**	***p*****value**	**Focus genes**
**Diseases and disorders**			
1	Cancer	2.09E−13 to 2.16E−03	1795
2	Infectious diseases	1.50E−22 to 1.50E−05	754
3	Renal and urological diseases	8.10E−18 to 8.01E−05	228
4	Organismal injury and abnormalities	1.62E−18 to 1.62E−18	164
5	Dermatological diseases and conditions	4.43E−18 to 4.43E−18	163

**Molecular and cellular functions**			
1	Cell death and survival	7.64E−18 to 2.46E−03	1427
2	Gene expression	2.37E−20 to 2.51E−04	1013
3	Cell cycle	2.82E−22 to 2.57E−03	801
4	DNA replication, recombination, and repair	8.36E−23 to 2.46E−03	648
5	RNA post-transcriptional modifications	1.22E−37 to 9.81E−04	214

**Physiological system development and function**			
1	Connective tissue development and functions	2.64E−10 to 2.46E−03	333
2	Embryonic development	3.37E−12 to 1.73E−03	323
3	Tissue morphology	8.40E−07 to 2.39E−03	255
4	Organismal development	2.27E−07 to 1.73E−03	220
5	Tissue development	6.03E−08 to 2.07E−03	119

**Table 2:** Top 10 significantly changed canonical signaling pathways between CRC patients and normal controls.

**Canonical pathway**	**−log (*****p*****value)**
EIF2 signaling	3.03E01
Protein ubiquitination pathway	2.38E01
Regulation of eIF4 and p70S6K signaling	1.64E01
Hereditary breast cancer signaling	1.19E01
Role of BRCA1 in DNA damage response	1.16E01
Mitotic roles of polo-like kinase	1.13E01
tRNA charging	1E01
mTOR signaling	9.18E00
Cell cycle control of chromosomal replication	9.02E00
Role of CHK proteins in cell cycle checkpoint control	8.09E00
